# Tumor Cell-Intrinsic BTLA Receptor Inhibits the Proliferation of Tumor Cells via ERK1/2

**DOI:** 10.3390/cells11244021

**Published:** 2022-12-12

**Authors:** Tian-You Cheng, Ya-Juan Liu, Hong Yan, Yi-Bo Xi, Li-Qiang Duan, Yang Wang, Tian-Tian Zhang, Yin-Min Gu, Xiao-Dong Wang, Chang-Xin Wu, Shan Gao

**Affiliations:** 1 Shanxi Academy of Advanced Research and Innovation, Taiyuan 030032, China; 2 Zhongda Hospital, Medical School, Advanced Institute for Life and Health, Southeast University, Nanjing 210096, China; 3 Suzhou Institute of Biomedical Engineering and Technology, Chinese Academy of Sciences, Suzhou 215163, China; 4 Institutes of Biomedical Sciences, Shanxi University, Taiyuan 030006, China; 5 Zhongda Hospital, School of Life Sciences and Technology, Advanced Institute for Life and Health, Southeast University, Nanjing 210096, China

**Keywords:** immune checkpoint, tumor cell-intrinsic BTLA, tumor cell-intrinsic HVEM, tumor suppressor, therapeutic target

## Abstract

B and T lymphocyte attenuator (BTLA) is an immune checkpoint molecule that mediates the escape of tumor cells from immunosurveillance. Consequently, BTLA and its ligand herpesvirus entry mediator (HVEM) are potentially immunotherapeutic targets. However, the potential effects of BTLA on tumor cells remain incompletely unknown. Here, we show that BTLA is expressed across a broad range of tumor cells. The depletion of BTLA or HVEM promotes cell proliferation and colony formation, which is reversed by the overexpression of BTLA in BTLA knockout cells. In contrast, overexpression of BTLA or HVEM inhibits tumor cell proliferation and colony formation. Furthermore, the proliferation of a subpopulation with high BTLA was also significantly slower than that of the low BTLA subpopulation. Mechanistically, the coordination of BTLA and HVEM inhibits its major downstream extracellular regulated protein kinase (ERK1/2) signaling pathway, thus preventing tumor cell growth. This study demonstrates that tumor cell-intrinsic BTLA/HVEM is a potential tumor suppressor and is likely to have a potential antagonist for immunotherapy, thus representing a potential biomarker for the optimal cancer immunotherapeutic treatment.

## 1. Introduction

Cancer is one of the leading causes of death worldwide [1]. The pathogenesis of tumor is coordinated with its genetic mutation and the ability to escape immune surveillance [2,3]. Thus, targeting immune checkpoints has become an attractive approach for immune therapy. Some agents such as targeting PD-1 or CTLA-4 have achieved certain clinical effects [4,5,6]. However, many cancer patients do not respond to such agents, or develop hyperprogressive disease (HPD) and pseudoprogressive disease (PPD) by various mechanisms [7,8,9], one of which is that tumor cell-expressing PD-1 potentially mediates HPD or PPD [10,11,12]. Therefore, a detailed study of immune checkpoints on tumor cells needs to be explored.

In addition to PD-1 and CTLA-4, BTLA is also an immune checkpoint, and is ligated by HVEM to inhibit T cell activation and survival [13,14,15,16], hence BTLA blockade can enhance cancer immunotherapy [17,18]. However, it has been shown that BTLA is expressed on ovarian cancer [19], gastric cancer [20,21], and non-small-cell lung cancer [22]. The potential function and mechanism of BTLA on tumor cells have not been elucidated. Moreover, HVEM plays an important role in antitumor, protumor function, or immune escape in some cancer types [21,23,24,25]. It has also been shown that HVEM has a broad expression on tumor cells [26,27,28]. However, the underlying mechanism and coordination of HVEM and BTLA on tumor cells remain elusive.

Clearly, the molecular understanding of BTLA is mainly confined to the interaction between the immune cells and tumor cells [25]. Here, we found that tumor cells broadly express both BTLA and HVEM. The silencing of BTLA or its ligand HVEM promotes cell proliferation via the ERK1/2 pathway, suggesting that tumor cell-intrinsic BTLA/HVEM is a potential tumor suppressor and is likely to have a potential antagonist for immunotherapy.

## 2. Materials and Methods

### 2.1. Cell Lines

NCI-H1299 and A549 cell lines were cultured in RPMI1640 medium (Gibco, made in Beijing, China) with 10% fetal bovine serum (FBS, Hyclone, Logan, UT, USA) and 1% penicillin/streptomycin (Hyclone). The HEK293T cell line was maintained in DMEM (Gibco) with 10% FBS and 1% penicillin/streptomycin (Hyclone). These cell lines were purchased from the Shanghai Cell Bank Type Culture Collection Committee, verified by short tandem repeat assays for their identification, and cultured at 37 °C in a humidified incubator with 5% CO_2_. These cell lines were tested for *Mycoplasma* contamination every month.

### 2.2. Flow Cytometric Analysis and Flow Cytometry Sorting

Cell surface expression of BTLA was detected by flow cytometry. Cells were collected and suspended in phosphate-buffered saline (PBS) containing of 0.5% bovine serum albumin (BSA) after being cultured for 48 h. The cell density was adjusted to 1 × 10^6^ cells/vial and these cells was blocked using human TruStain FcX (Biolegend, San Diego, CA, USA) for 10 min. Then, cells were incubated with the directly labeled antibody of BTLA protected from light at 4 °C for 1 h. Then, the cells were washed with PBS twice and were filtered with a filter for analysis or cell sorting. A total of 30,000 cells were analyzed on BD FACS Celesta (BD biosciences, San Jose, CA, USA) using the CELL Quest software and a total of 1 × 10^7^ cells were sorted on a Beckman FACS Celesta (Beckman, Pasadena, CA, USA). Images were analyzed by FlowJo software version 10.4 (FlowJo, LLC, Ashland, OR, USA).

### 2.3. Cell Titer-Glo Luminescent Cell Viability Assay

Cells were plated in 96-well plates with a density of 1–2 × 10^3^ cells per well. Cell proliferation was measured with a Cell Titer-Glo Luminescent Cell Viability (CTG) Assay Kit (Promega, Madison, WI, USA) using a multimode microplate reader (Synergy HTX, BioTek, Winooski, VT, USA) and the quantified data of each group were normalized to the corresponding 0 d value to calculate the relative cell proliferation. All experiments were performed in three independent biological experiments.

### 2.4. Immunoblot

The total cell lysates were extracted using the radio immunoprecipitation assay (RIPA) lysis buffer (Beyotime Biotechnology Inc., Jiangsu, China) containing 1% protease and phosphatase inhibitor cocktails (Sigma, St. Louis, MO, USA). Protein concentration was measured by the BCA Protein Assay Kit (Beyotime Biotechnology Inc.). Equal protein was separated by SDS–PAGE and transferred to a polyvinylidenefluoride (PVDF) membrane (Millipore, made in Tullagreen, Ireland). The membrane was blocked for 1 h in TBST containing 0.1% Tween-20 with 5% non-fat milk followed by overnight incubation with the primary antibody diluted at 4 °C. After adequate washing, the membrane was incubated with HRP-conjugated secondary antibody (EASYBIO, Seoul, Korea) at room temperature for 1 h in TBST containing 5% non-fat milk, followed by extensive washing and detection. BTLA, p-ERK, ERK, and GAPDH were detected by immunoblot. Among them, the detection steps of HVEM were optimized according to [29]. Images were obtained with Armersham Image 600 (GE Health-care, Chicago, IL, USA). The intensities of the blots were analyzed using ImageJ software (NIH, Bethesda, MD, USA), and the quantified data of each phosphorylated protein were normalized to the corresponding total protein.

### 2.5. qRT-PCR

Total RNA was isolated using RNAiso Plus (Takara, made in Beijing, China) and the RNA was subjected to reverse transcription by the PrimeScript™ RT Reagent Kit with gDNA Eraser (Takara). Then, RT-PCR was performed and qRT-PCR was conducted using SYBR premix EX Taq (Takara) and analyzed with a QuantStudio 7 Flex Real-Time PCR System (Thermo Fisher, Waltham, MA, USA), following the manufacturer’s instructions. The cycle threshold values (CT) numbers were recorded, which were normalized against an internal control (*GAPDH*). The relative gene expression levels were analyzed using the 2-ΔΔCT method [30]. The primers used in all qRT-PCR assays are listed in Supplementary Table S1 including *GAPDH* [31].

### 2.6. Public ChIP Sequence Data Collection

The H3K27Ac ChIP-sequence data in the GM12878, hESC, HSMM, HUVEC, K562, NHEK, and NHLF cell lines and the H3K4me1 ChIP-sequence data in the HepG2 cell line were both sourced from GSE29611 [32]. The enhancer data were sourced from the url: https://genome.ucsc.edu/cgi-bin/hgTrackUi?db=hg19&g=geneHancer, accessed on 28 September 2022.

### 2.7. RNA-Sequence and Analysis

RNA-sequence of BTLA knockdown (KD) vs. the control cells and the sorting subpopulation of low BTLA expression vs. high BTLA subpopulation were performed and analyzed by Berry Genomics (Beijing, China). Raw data were filtered to remove the adaptors and low-quality bases using trim galore (version 0.6.6). The obtained fastq files were then aligned using STAR (version 2.7.0d) with default parameters to the human reference transcriptome GRCh38.97 from Ensemble. Gene counts were obtained using the feature Counts package (version 2.0.1) within the Subread package, and edge R package (version 3.32.1) was used to identify differentially expressed genes between BTLA KD vs. the control, and the subpopulation of the low BTLA vs. high BTLA subpopulation [33]. Genes with *p*-value ≤ 0.05 and log2 Fold change (FC) ≥ 1.0 were counted as the upregulated genes, and genes with *p*-value ≤ 0.05 and log2 Fold change (FC) ≤ −1.0 were counted as the downregulated genes. Data were visualized using the ggplot2 R package. The RNA sequencing data from this study have been deposited in the NCBI Gene Expression Omnibus database (GSE207471).

### 2.8. Expression Vectors, shRNAs, and Small Guide RNA (sgRNA)

To generate lentiviruses expressing the indicated shRNAs or indicated genes, HEK293T cells were transfected with shRNAs cloned in vector pSIH-H1-Puro or genes with 3 × Flag at the 3′-terminus cloned into the vector pLVX-IRES-Neo, PMD2.G, and PSPAX2 with a ratio of 8:6:3 using 1 mg/mL polyethyleneimine (PEI, YESEN, made in Shanghai, China) with a ratio of 1:3 (m/m). The pSIH-H1-Puro and pLVX-IRES-Neo empty vectors were used for the control, respectively. After transfection with 6 h, cells were cultured in fresh medium with 10% FBS for an additional 48–72 h. The culture medium containing the lentivirus particle was centrifuged at 4000× *g* for 10 min, and about 3 mL cleared supernatant was added into a 60 mm Petri dish with 50–70% confluence cells for infection. After that, cells infected with lentivirus were selected in either 1.0–2.0 µg/mL puromycin (Puromycin Dihydrochloride, Life Technologies, Carlsbad, CA, USA) and/or 500–700 µg/mL neomycin (G418 sulfate, Life Technologies). The effective lentivirus-mediated KD and overexpression (OE) were verified by qRT-PCR or immunoblot analysis in each experiment. The BTLA KO NCI-H1299 cell was generated by CRISPR/Cas9 and the sgRNA (5′-GTGAAATACTGTGCTAACAG-3′) was cloned into Vector LentiCRISPRv2 [34]. The KO efficiency of BTLA was confirmed by immunoblot and DNA sequence. The shRNA sequences are shown in Supplementary Table S2.

### 2.9. Clone Formation Assay

About 500 or 1000 cells were plated in 6-well plates per well and incubated for 10 days at 37 °C in a humidified incubator with 5% CO_2_. Colonies were stained with crystal violet staining solution (0.5% (*w*/*v*) crystal violet with PBS) after being fixed with 95% (*v*/*v*) ethanol, and then counted. All experiments were performed in triplicate.

### 2.10. Antibodies

Antibodies used are listed in Supplementary Table S3.

### 2.11. Statistical Analysis

Data were all presented as the mean ± standard deviation (SD). Comparisons for gene levels, colony formation numbers, and relative cell proliferation were performed using two-tailed unpaired Student’s *t* test. Spearman’s correlation was performed to analyze the correlation of RNA between BTLA and HVEM in LUSC. *p* value of <0.05 was considered statistically significant. All statistical analyses and graph plotting were performed with GraphPad Prism 6.

## 3. Results

### 3.1. BTLA Is Expressed by a Subpopulation of Tumor Cells

**Figure 1:**
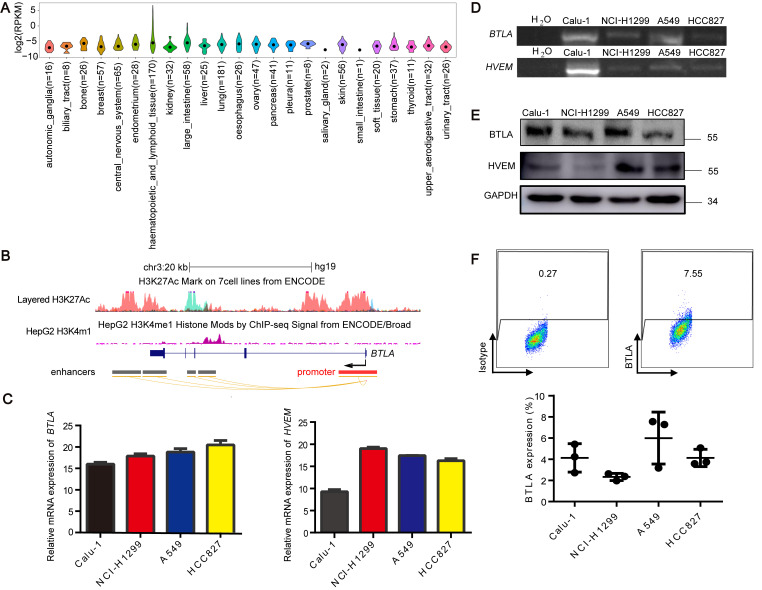
BTLA is expressed by tumor cells. (**A**) Violin plot showing expression levels of BTLA in various kinds of cancer cells based on data from Cancer Cell Line Encyclopedia (CCLE). The black dot indicates the median. (**B**) The UCSC genome browser view of the relative binding enrichments of H3K27Ac and H3K4me1 around the BTLA gene. (**C**–**E**) qRT-PCR (delta CT for gene transcript level and normalized to corresponding GAPDH) for BTLA (left) and HVEM (right) (**C**), RT-PCR (**D**) and immunoblot analysis (**E**) for BTLA (top) and HVEM (bottom) in the indicated cancer cell lines. (**F**) Representative flow cytometry plots (top) and percentages (mean ± SD, bottom) of the BTLA surface protein expression on the indicated cancer cell lines (*n* = 3).

To explore whether BTLA is expressed on tumor cells, we analyzed the expression profile of BTLA in The Cancer Genome Atlas (TCGA) and found that BTLA was widely transcribed in 32 cancer tissue types (Supplementary Figure S1A). However, these data may be affected by infiltrating lymphocytes in the isolated tissues. Therefore, we analyzed BTLA transcription in the Cancer Cell Line Encyclopedia (CCLE) with pure cancer cell lines [35], which revealed that BTLA was transcribed in cancer cell lines (Figure 1A). Furthermore, we observed that two epigenetic signatures of H3K27Ac and H3K4me1 associated with the activation of transcription were enriched around the transcriptional start sites of BTLA in the Encyclopedia of DNA Elements (ENCODE) (Figure 1B) [36]. H3K4me1 and H3K27Ac are also associated with a gene transcriptional enhancer, therefore, we further analyzed enhancers within BTLA regulated by the two epigenetic signatures [37]. Indeed, we identified the potential enhancers associated with BTLA, which were enriched by H3K4me1 and H3K27Ac. Altogether, these data suggest that BTLA is transcribed by cancer cells.

To further verify the above findings, we examined the BTLA expression in different cancer cell lines that were used in our lab including 22 cell lines representing six kinds of cancers. Real-time quantitative reverse transcription polymerase chain reaction (qRT-PCR) and RT-PCR for these cancer cells demonstrated that BTLA messenger RNA (mRNA) was transcribed in all examined cancer cell lines (Figure 1C,D and Figure S1B). Immunoblot analysis revealed that BTLA was expressed by non-small cell lung cancer (NSCLC) with the size about 55 KDa, which is similar to the size of T cell-intrinsically expressed BTLA (Figure 1E) [13]. Moreover, we evaluated the BTLA expression by flow cytometry and the results showed that BTLA was expressed in a subpopulation (Figure 1F) (BTLA positively ranged from 1.97% to 7.55% in lung cancer cell lines). It has been shown that HVEM is the ligand of BTLA, and the ligation can lead to the inhibition of T cell activation and immune escape of tumor cells [13,15]. Therefore, we also investigated whether HVEM is expressed on these tumor cells. As expected, HVEM was expressed on these cancer cell lines (Figure 1C–E and Figure S2A,B). Indeed, we also found a weak but positive correlation between BTLA and HVEM of NSCLC in TCGA data (Supplementary Figure S2C). BTLA and HVEM were also expressed in clinical cancer samples [20,22], supporting our findings. Overall, these data demonstrate that BTLA is expressed in a subpopulation of cancer cells.

### 3.2. BTLA Inhibits the Growth of Tumor Cells via ERK1/2 Pathway

**Figure 2:**
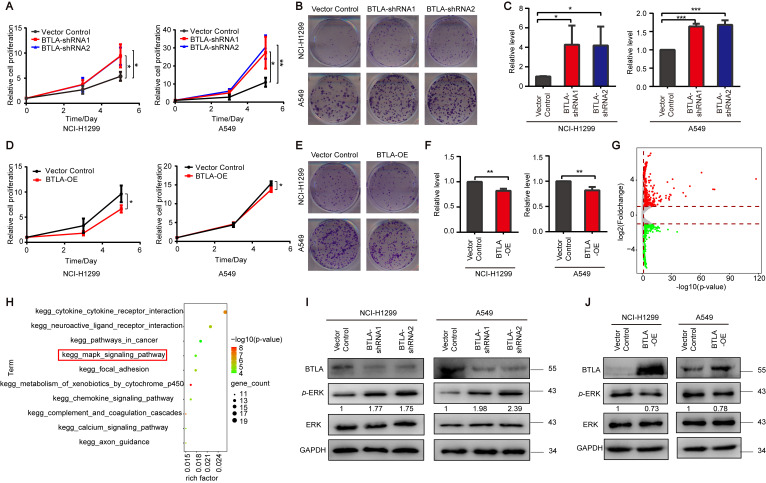
Inhibition of tumor cell growth by tumor cell-intrinsic BTLA. (**A**,**D**) The Cell Titer-Glo Luminescent Cell Viability (CTG) assay for the cell proliferation of NCI-H1299 (left) and A549 (right) cells transfected with the indicated shRNAs (**A**) or plasmids (**D**) (*n* = 3). (**B**,**E**) Representative images of colony formation for NCI-H1299 (top) and A549 (bottom) cells transfected with the indicated shRNAs (**B**) or plasmids (**E**) (*n* = 3). (**C**,**F**) Quantification data of colony formation for NCI-H1299 (left) and A549 (right) transfected with the indicated shRNAs (**C**) or plasmids (**F**) (*n* = 3). (**G**) Volcano plot showing differentially expressed genes (DEGs) in BTLA KD of A549 cells. (**H**) The top 10 enriched KEGG pathways of BTLA KD enriched by DEGs from (**G**). (**I**,**J**) Immunoblot analysis of the indicated proteins in NCI-H1299 (left) and A549 (right) cells transfected with the indicated shRNAs (**I**) or plasmids (**J**). Data are presented as the mean ± SD from three independent experiments. * *p* < 0.05, ** < 0.01, *** < 0.001.

To functionally dissect the potential role of tumor-intrinsic BTLA in tumor cells, we knocked down endogenous BTLA using two short hairpin RNAs (shRNAs) that target the distinct sequences in NCI-H1299 and A549 cells (Supplementary Figure S3A), which led to a significant increase in cell proliferation and colony formation compared to the control, respectively (Figure 2A–C). Moreover, we generated two BTLA knockout (KO) cell lines in NCI-H1299 using CRISPR-Cas9 techniques (Supplementary Figure S4A,B), which also resulted in the increased cell proliferation (Supplementary Figure S4C). Conversely, we overexpressed BTLA in both cell lines, as indicated by the inhibition of cell proliferation and colony formation (Figure 2D–2F) and observed increases in both the mRNA and protein levels (Figure 2J and Figure S3B).

As BTLA regulates several downstream signaling pathways including PI3K/AKT, NF-κB, and ERK1/2 in T or B cells [15,16,24,38], and to further examine whether these signaling pathways are regulated by BTLA expressed on tumor cells, we performed the RNA sequencing (RNA-Seq) of BTLA knockdown (KD) cells. The gene expression profile after BTLA KD showed the downregulation of 648 genes and upregulation of 782 genes, which were indeed enriched in cell growth-related pathways including mitogen-activated protein kinase (MAPK) signaling pathway (Figure 2G,H and Figure S5A), according to a Kyoto Encyclopedia of Genes and Genomes (KEGG) database analysis [39]. Therefore, we hypothesized that BTLA inhibits tumor growth by inactivating the ERK1/2 pathway in cancer cells. To test this hypothesis, we further validated the results by immunoblot analysis in the BTLA KD and KO cancer cells. The data showed that the phosphorylation of ERK1/2 (p-ERK1/2) was increased in the BTLA KD and KO cells compared to the control cells, respectively (Figure 2I and Figure S4D). Correspondingly, cells with the overexpression of BTLA showed a decrease in the p-ERK1/2 level compared to the control (Figure 2J). Moreover, we observed that cell proliferation was attenuated and the level of p-ERK1/2 was downregulated when BTLA was expressed in the BTLA KO cell lines (Supplementary Figure S4E,F). Altogether, these data suggest that tumor cell-intrinsic BTLA is a potential tumor suppressor that deregulates the ERK1/2 signaling pathway.

### 3.3. The Subpopulation of Low BTLA Grows Faster than High BTLA Subpopulation

**Figure 3:**
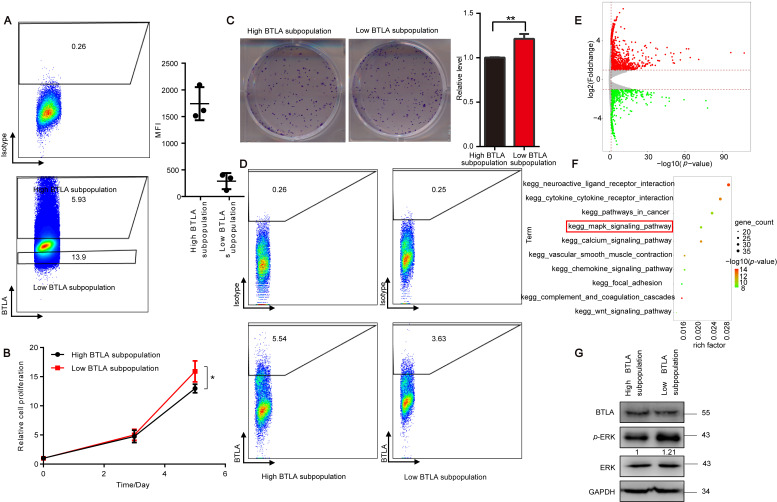
Differences between the subpopulation of BTLA^low^ and BTLA^high^. (**A**) Representative images of cell sorting (left). Quantitation of BTLA protein levels in high and low BTLA subpopulations collected by cell sorting is shown as the mean fluoresce intensity (MFI) (right) (*n* = 3). (**B**) The CTG assay assessing the cell proliferation of BTLA^high^ subpopulation and BTLA^low^ subpopulation cells (*n* = 3). (**C**) Representative images of colony formation (left) and quantification data (right) for high and low BTLA subpopulation cells (*n* = 3). (**D**) Representative flow cytometry plots of high and low subpopulations cells cultured for 10 days after cell sorting. (**E**) Volcano plot showing DEGs in two subpopulations collected by the cell sorting of A549 cells. (**F**) The top 10 enriched KEGG pathways of two subpopulations enriched by DEGs from (**E**). (**G**) Immunoblot analysis of high and low BTLA subpopulation cells. Data are presented as the mean ± SD from three independent experiments. * *p* < 0.05, ** < 0.01.

To further confirm the effect of tumor cell-intrinsic BTLA on the tumor cells, flow cytometric cell sorting was used to generate BTLA^high^/BTLA^low^ lung cancer subpopulations labeled with BTLA monoclonal antibody, which is an important method to study tumor cell subpopulations [11,12]. The sorting results using mean fluorescence intensity showed that two subpopulations with different expression levels of BTLA were indeed sorted (Figure 3A). We next compared the cell growth between the BTLA^high^ and BTLA^low^ subpopulations sorted A549 cells and found that BTLA^low^ subpopulations had increased proliferation and colony formation compared to the BTLA^high^ cells (Figure 3B,C). We also performed cell culture in vitro and RNA-Seq of the sorted subpopulations, and found that BTLA expression was still different between the two subpopulations after 10 days of culture, but the difference between the two subpopulations was reduced, not as large as we expected (Figure 3D). We speculated that this narrowing of differences is likely due to the convergence of tumor cells during post-sorting culture, as is the convergence of subpopulations after stem cell sorting [40]. We then analyzed the RNA-Seq data of the two selected subpopulations and gene expression profile revealed the downregulation of 886 genes and upregulation of 1562 genes in BTLA^low^ subpopulation compared with BTLA^high^ subpopulation, and as expected, the MAPK pathway was enriched by KEGG analysis (Figure 3E,F and Figure S5B). A similar result was found for the protein level of p-ERK1/2 between the two subpopulations (Figure 3G). Collectively, these data indicate that there is difference between different BTLA expression subpopulations, and tumor-intrinsic BTLA is a potential tumor suppressor.

### 3.4. HVEM Inhibits the Growth of Tumor Cell via ERK1/2 Pathway

**Figure 4:**
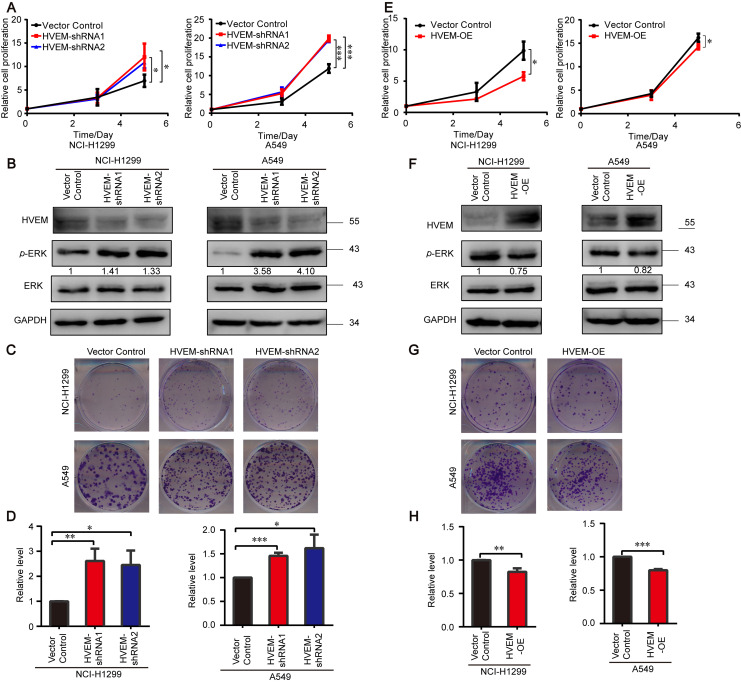
Inhibition of tumor cell growth by tumor cell-intrinsic HVEM. (**A**,**E**) The CTG assay assessing the cell proliferation of NCI-H1299 (left) and A549 (right) cells transfected with the indicated shRNAs (**A**) or plasmids (**E**) (*n* = 3). (**B**,**F**) Immunoblot analysis of the indicated proteins in cells transfected with the indicated shRNAs (**B**) or plasmids (**F**). (**C**,**G**) Representative images of colony formation for NCI-H1299 (top) and A549 (bottom) transfected with the indicated shRNAs (**C**) or plasmids (**G**) (*n* = 3). (**D**,**H**) Quantification data of colony formation for NCI-H1299 (left) and A549 (right) cells transfected with the indicated shRNAs (**D**) or plasmids (**H**) (*n* = 3). Data are presented as the mean ± SD from three independent experiments. * *p* < 0.05, ** < 0.01, *** < 0.001.

HVEM is the only reported ligand for BTLA and is expressed on tumor cells to mediate immune tolerance, and the ligation of BTLA and HVEM can negatively regulate the proliferation of lymphocytes [14,41]. Thus, we hypothesized that HVEM has a similar function as BTLA in tumors. To confirm this hypothesis, we first knocked down endogenous HVEM using two shRNAs that target the different sequence of HVEM in the NCI-H1299 and A549 cells. The results revealed that the rates of cell proliferation and colony formation in the HVEM KD cells were significantly increased in comparison with the control cells accompanied by the decrease in the mRNA and protein level in the HVEM KD cells compared to the control (Figure 4A–D and Figure S3C). Consistently, HVEM KD enhanced the level of p-ERK1/2 compared to the control cells (Figure 4B). In contrast, cell proliferation and colony formation were inhibited in the HVEM-overexpressed cells compared to the control when we observed increased mRNA and protein levels after overexpressing HVEM in both cell lines (Figure 4E–H and Figure S3D). Indeed, p-ERK1/2 was downregulated in the HVEM-overexpressed tumor cells (Figure 4F). Overall, these data demonstrate that HVEM inhibits tumor cell growth and inactivates ERK1/2.

### 3.5. BTLA/HVEM Axis Functions on Growth and Signaling Pathway in Cancer Cells

**Figure 5:**
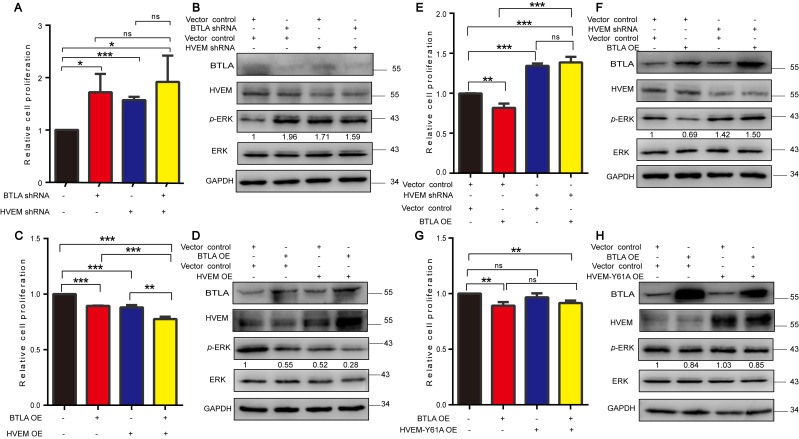
The effects of BTLA depend on HVEM. Relative cell proliferation (**A**,**C**,**E**,**G**) (*n* = 3) and immunoblot analysis (**B**,**D**,**F**,**H**) for cells expressing the indicated shRNAs or plasmids. Data are presented as the mean ± SD from three independent experiments. * *p* < 0.05, ** < 0.01, *** < 0.001, ns, not significant.

Given that BTLA is engaged by HVEM in lymphocytes and follicular lymphoma [14,25], as the similarly observed effects of BTLA and HVEM on cancer cells, we further investigated whether tumor cell-intrinsic BTLA was also engaged by HVEM to regulate the cell growth and signaling pathway. We first explored the effects of the simultaneous KD of BTLA and HVEM expression in NCI-H1299 cells. Immunoblot confirmed KD efficiencies and found that all KD cells showed increased cell proliferation compared to the control cells, but the simultaneously double KD of both did not further increase proliferation compared to either BTLA or HVEM KD alone (Figure 5A,B). Consistently, KD of either BTLA or HVEM expression enhanced the p-ERK1/2 level, but the simultaneously double KD of both did not further enhance the p-ERK1/2 level (Figure 5B). Since BTLA and HVEM were expressed in a subpopulation of cancer cells, we hypothesized that cell growth may further be inhibited by the simultaneous overexpression of BTLA and HVEM. Indeed, cells with the simultaneous overexpression of BTLA and HVEM exhibited significantly decreased proliferation compared to cells overexpressed with either BTLA, HVEM, or the control (Figure 5C,D). Consistently, simultaneous transfection with both BTLA and HVEM significantly suppressed the p-ERK1/2 level compared to the overexpression of either BTLA or HVEM alone (Figure 5D). These data indicate that BTLA and HVEM are an axis in regulating cell proliferation.

Next, we explored whether the BTLA transduction signaling pathway was HVEM-dependent in NCI-H1299 cells. Thus, we overexpressed BTLA in those cells in which HVEM was KD by HVEM-specific shRNA. The results showed that overexpression of BTLA in the HVEM KD cells abolished the inhibitory effect of BTLA on tumor cells (Figure 5E,F). As BTLA engages HVEM in its first cysteine-rich domain (CRD1) and mutation at the 61 tyrosine to alanine (Y61A) of the HVEM CRD1 domain disrupts the binding to BTLA [13,42,43], we hypothesized that Y61A HVEM could not play a regulatory role in the tumor cell. Indeed, the overexpression of Y61A HVEM had no effects on cell proliferation compared to the control, and cells with simultaneous overexpression of BTLA and Y61A HVEM could not further reduce proliferation compared to cells overexpressing BTLA (Figure 5G). Consistently, the overexpression of Y61A HVEM did not affect the p-ERK1/2 level compared to the control, and cells with simultaneous overexpression of BTLA and Y61A HVEM had almost the same effect on the p-ERK1/2 level as cells overexpressed BTLA (Figure 5H), suggesting that HVEM is required for BTLA signaling. Collectively, these data demonstrate that the BTLA/HVEM axis coordinates to regulate the cell proliferation and signaling pathway in tumor cells.

## 4. Discussion

BTLA is a coinhibitory molecular that is structurally and functionally related to CTLA-4 and PD-1 [44], and is mainly expressed on T cells, B cells, dendritic cells, and myeloid cells [45]. CTLA-4 and PD-1 are expressed in human solid tumor-derived cells [10,12,46,47], and recent studies have shown that BTLA is expressed in a small subpopulation of cancer cells from clinical tumor specimens mainly identified by immunohistochemical including NSCLC [22], gastric cancer [20], and ovarian cancer cells [19]. The high expression of BTLA in gastric cancer tissue is associated with poor prognosis such as lymph node metastasis and tumor invasion [21]. In this study, we demonstrated that BTLA is transcribed in various cancer cell lines and further confirmed the BTLA expression by RT-PCR, immunoblot, and flow cytometry. In addition, we confirmed that HVEM is also expressed on tumor cells. Thus, this study clearly revealed that tumor cells contain BTLA and HVEM positive subpopulations. Given these observations, we even speculate that most immune checkpoint molecules are likely to be expressed in a subpopulation of tumor cells, but this requires more research in the future.

To date, the function of BTLA has been mainly studied in T cells [15,48]. In the tumor-infiltrating lymphocytes (TILs), the BTLA^low^ CD8+ TIL subpopulation grows faster than the BTLA^high^ CD8+ TIL subpopulation, but BTLA^high^ CD8+ TILs display improved survival following the killing of a tumor target and heightened “serial killing” capacity, and the gene expression profile is associated with T-cell tolerance [15,16,25]. Tumor cell-intrinsic BTLA has a protumor effect on ovarian cancer, which could counteract the effects of miRNA-32 [19]. It has been shown that the increased BTLA and HVEM levels correlate with the development and poor prognosis of gastric cancer patients [20,21], and that high BTLA expression may predict prognosis in patients in NSCLC [22]. These data suggest that tumor cell-intrinsic BTLA is a potential oncogene. Tumor cell-intrinsic HVEM has also been shown to contribute to tumor progression and poor prognosis and play a protumor effect [26,49]. However, a functional study showed that loss of HVEM or BTLA leads to cell autonomous activation of B cell proliferation and drives the development of germinal center lymphomas in vivo [24]. Consistently, our results showed that BTLA expressed by NSCLC cells inhibits tumor growth in vitro. Functional assays showed that KD or KO of endogenous BTLA or HVEM significantly enhanced the proliferation and colony formation of NSCLC cells, whereas the overexpression of BTLA or HVEM markedly reduced the proliferation and colony formation. Furthermore, we sorted BTLA^high^ and BTLA^low^ lung cancer subpopulations and found that the proliferation of a subpopulation with high BTLA was also significantly slower than that of the low BTLA subpopulation. Our results support the hypothesis that BTLA plays a crucial role in NSCLC progression and suggest that the functional role of BTLA is being extended into nonimmune cell types, but these findings require more in vivo experimental evidence to be further validated. Together, these data also suggest that BTLA may function across a broad range of tumor types, and tumor cell-intrinsic BTLA plays an antagonist function in different tumor types/cell lines. This antagonist switching in tumor cell function may be defined as the “tumor cell intrinsic BTLA/HVEM paradox”, which is mediated by the different types of tumor cells or by selective signaling pathways, similar to the “tumor cell intrinsic PD-1/L1 paradox” [10]. Given the above results, we even speculate that a similar paradox may occur when other immune checkpoint proteins are expressed in tumors. However, we need more and deeper studies in more tumor types and models to further corroborate our experimental results. Altogether, these findings help to further understand the complex role of immune checkpoints on tumors and the benefit to cancer patients through the development of optimal immune checkpoint therapy.

BTLA ligation by HVEM acts in a multifaceted role with the singular ability to control cell growth and the production of interleukin 2 (IL-2) in CD8+ T cells [15], which regulates several downstream signaling pathways including PI3K/AKT, NF-κB, and ERK1/2 in T cells or B cells [15,16,24,38]. Our work confirmed that BTLA regulates cell growth by the activation of ERK1/2, which transduces signaling depending on its ligand HVEM, a similar result observed in lymphoma [24]. In addition, according to the latest clinical data, PD-1 pathway inhibitors have shown potential to treat multiple advanced cancer types. However, only a minority of cancer patients exhibited clinical responses to checkpoint antibodies [50]. It has been demonstrated that the BTLA signaling pathway is not the same as PD-1 [10,11,12], and our results also confirmed this view, making it a potential for combined targeted therapy with PD-1 or PD-L1 for translational importance. However, we still need more evidence to support this combination in the future. Furthermore, considering the similarity and antitumor effect of tumor cell-intrinsic BTLA function to PD-1 [10], we also speculate that when the BTLA monoclonal antibody is used to treat tumors, there may also be similar adverse effects such as PPD or HPD. Therefore, it is worth evaluating the preclinical efficiency of inhibitors targeting BTLA in the future.

## 5. Conclusions

**Figure 6:**
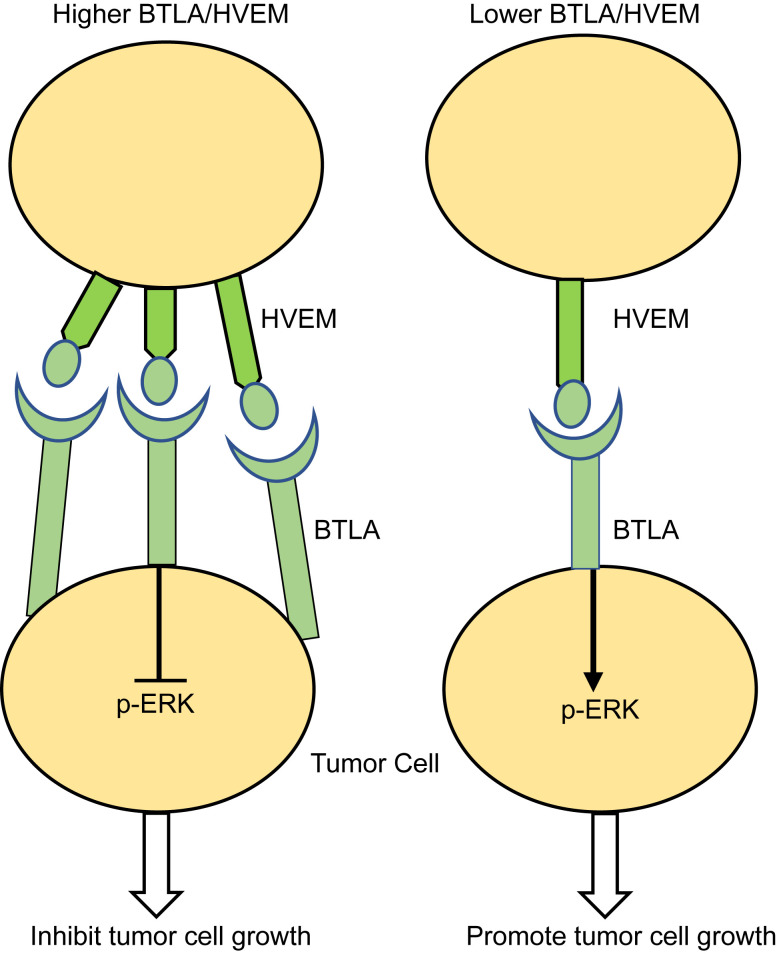
A proposed model for the BTLA/HVEM axis on tumor cell growth. Tumor cells express BTLA/HVEM, which inhibit tumor cell growth through the inactivation of the ERK1/2 signaling pathways.

Collectively, our data show the comprehensive characterization of BTLA transcription and protein expression in various tumors. The tumor cell-intrinsic BTLA/HVEM axis suppresses the tumor growth via the ERK1/2 pathway (Figure 6). As immune checkpoint therapy, especially targeting BTLA, is becoming widely available to cancer patients, it is critical to understand the underlying function and mechanism of these immune checkpoints, thus benefiting patients with advanced cancer.

## Data Availability

All data are available in the manuscript or the Supplementary Materials. RNA-Seq raw data have been deposited in the NCBI Gene Expression Omnibus database under accession number GSE207471.

## Supplementary Materials

The following supporting information can be downloaded at: https://www.mdpi.com/article/10.3390/cells11244021/s1, Figure S1: *BTLA* is transcribed in various tumor cells; Figure S2: *HVEM* is transcribed in various tumor cells; Figure S3: Relative *BTLA* and *HVEM* mRNA expression; Figure S4: The cell proliferation is reversed by overexpressing BTLA in BTLA KO cells; Figure S5: Heatmap showing the differentially expressed genes enriched in the MAPK signaling pathway in BTLA KD and sorting cells of A549; Table S1: List of qRT-PCR primers; Table S2: Sequences of shRNA for KD; Table S3: List of primary antibodies for WB and antibodies for FACS.
