# Supplementary material for: Tumor Cell-Intrinsic BTLA Receptor Inhibits the Proliferation of Tumor Cells via ERK1/2
Source: Cells. 2022 Dec 12;11(24):4021. doi: 10.3390/cells11244021 (PMC9777428; doi:10.3390/cells11244021)
Supplement: Supplementary file 1 [file cells-11-04021-s001.zip › cells-2042098-supplementary.pdf]

Supplementary Figures

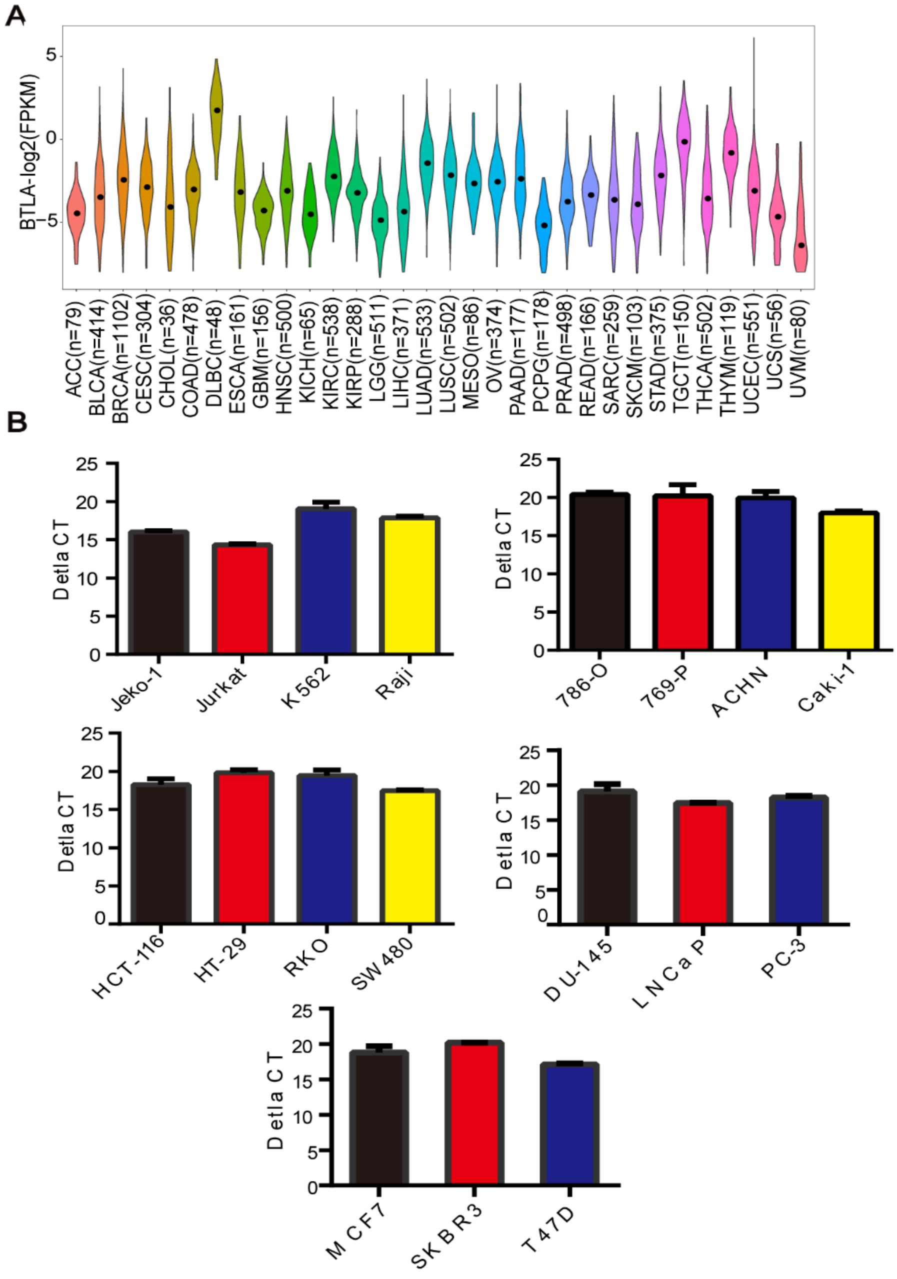

Figure S1. *BTLA* is transcribed in various tumor cells. (**A**) Violin plots showing the expression levels of *BTLA* in various kinds of clinical tumor tissues based on data from TCGA. Black dot indicates the median. (**B**) qRT-PCR expression analysis of *BTLA* mRNA in blood cancer, kidney cancer, colon cancer, prostate cancer and breast cancer cell lines (n = 2). Data are presented as the mean ± SD of two independent experiments.

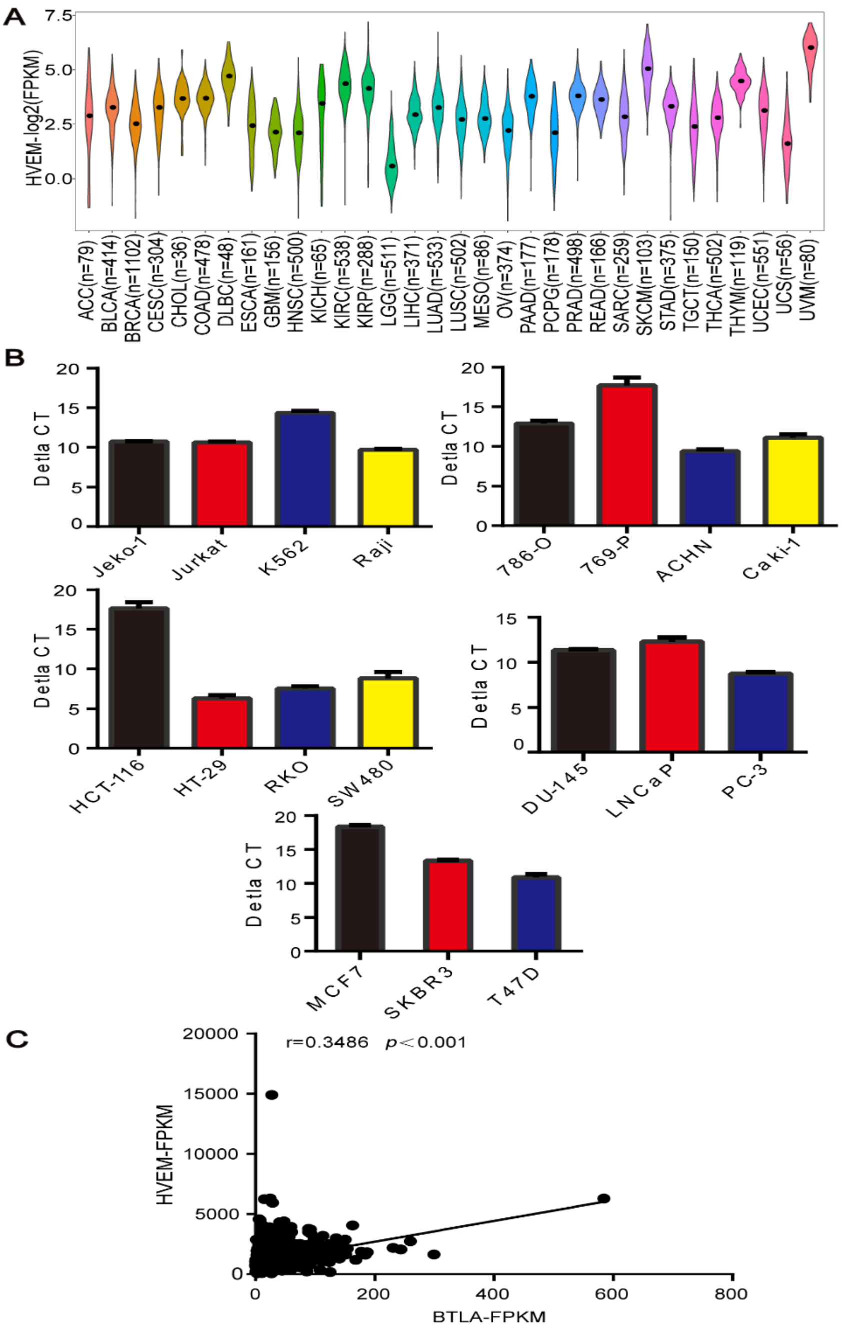

Figure S2. *HVEM* is transcribed in various tumor cells. (**A**) Violin plots showing the expression levels of *HVEM* in various kinds of clinical tumor tissues based on data from TCGA. Black dot indicates the median. (**B**) qRT-PCR expression analysis of *HVEM* mRNA in blood cancer, kidney cancer, colon cancer, prostate cancer and breast cancer cell lines (n = 2). (**C**) Correlation analysis of *BTLA* and *HVEM* in NSCLC on data from TCGA. Data are presented as the mean ± SD of two independent experiments.

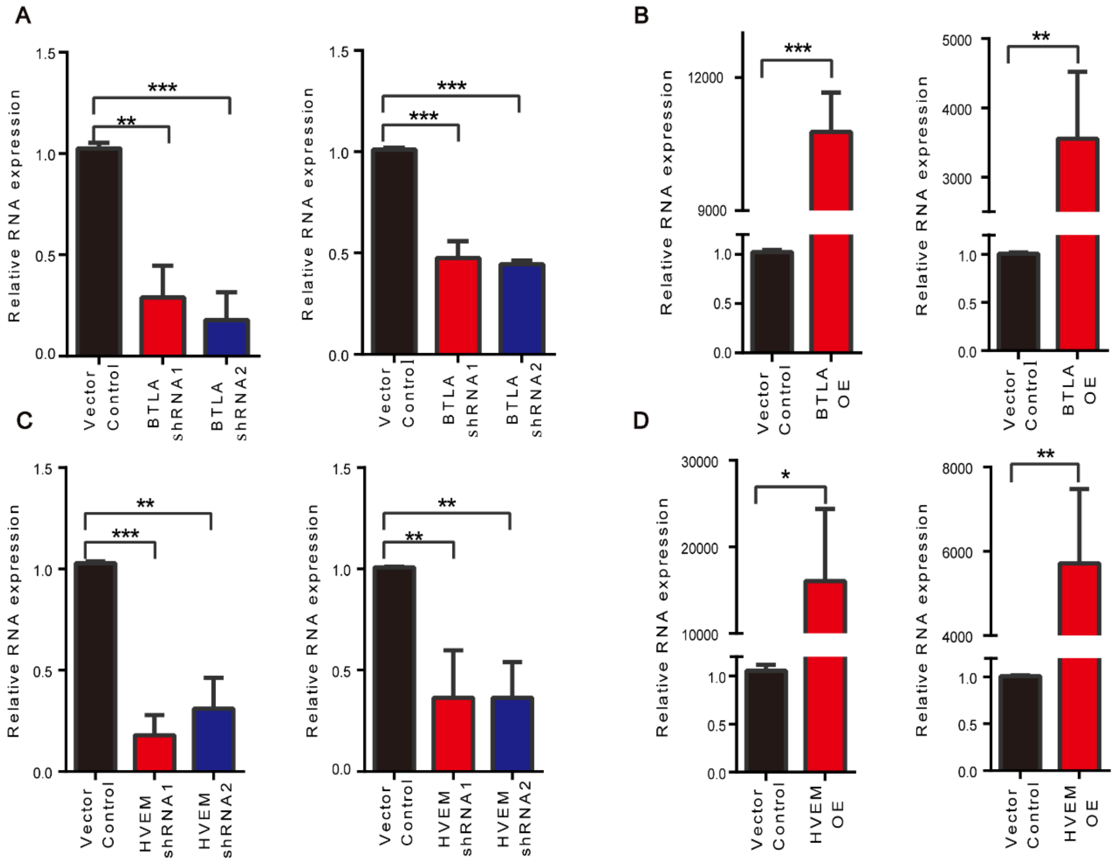

Figure S3. Relative *BTLA* and *HVEM* mRNA expression. (**A-D**) Quantification of qRT-PCR from cells (Left: NCI-H1299, Right: A549) transfected with the indicated plasmids (n = 3). *p < 0.05, **< 0.01, ***< 0.001.

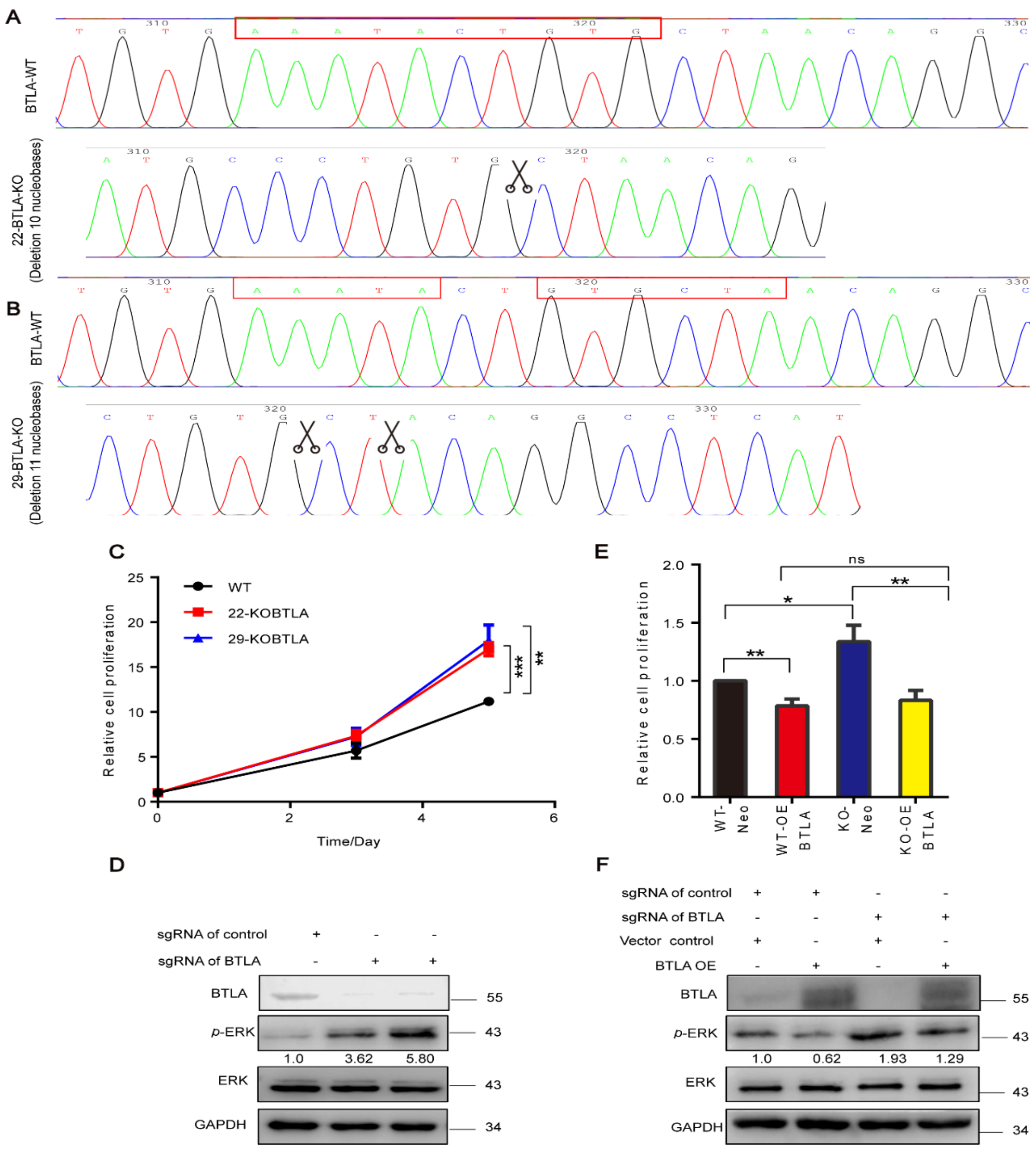

Figure S4. The cell proliferation is reversed by overexpressing BTLA in BTLA KO cells. (**A** and **B**) Genomic DNA sequence analysis of *BTLA* KO clones in NCI-H1299 cell using CRISPR-Cas9 techniques. The red boxes represent the sequence deleted of genomic DNA in the KO cell lines compared to the wild type. The scissors represent the starting position of the sequence deletion in the KO cell lines. (**C-F**) Relative cell proliferation (**C** and **E**) (n = 3) and immunoblot analysis (**D** and **F**) of the indicated proteins in NCI-H1299 cells with the indicated plasmids. *$p < 0.05$, **< 0.01, ***< 0.001.

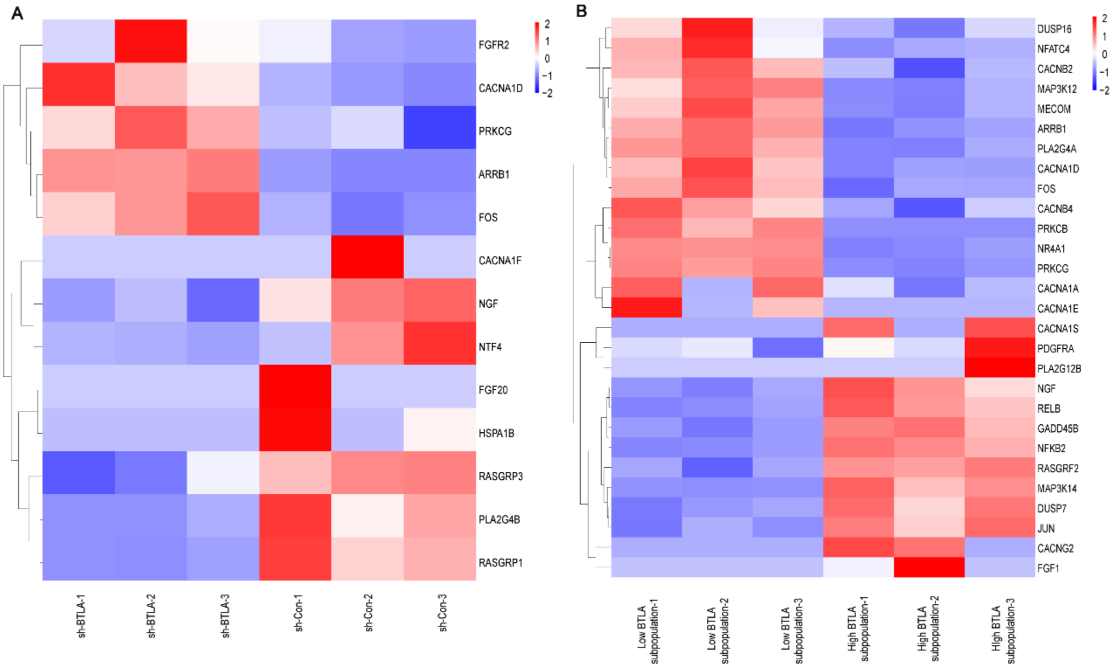

Figure S5. Heatmap showing the differentially expressed genes enriched in MAPK signaling pathway in BTLA KD (**A**) and sorting cells (**B**) of A549.

Supplementary Tables

Table S1. List of qRT-PCR primers

Genes	Forward (5’-3’)	Reverse (5’-3’)
BTLA	CATCTTAGCAGGAGATCCCTTTG	GACCCATTGTCATTAGGAAGCA
HVEM	GTGCAGTCCAGGTTATCGTGT	CACTTGCTTAGGCCATTGAGG
GAPDH[1]	GGAGCGAGATCCCTCCAAAAT	GGCTGTTGTCATACTTCTCATGG

Supplementary Table S2. Sequences of shRNA for KD

Targets	shRNA-Forward (5'-3')	shRNA-Reverse (5'-3')
sh-BTLA-1	GATCCCGCTATCAGAAACTGG AATTTATGATAATGATTCAAGA GATCATTATCATAAATTCCAGT TTCTGATAGTTTTTTG	AATTCAAAAAACTATCAGAAA CTGGAATTTATGATAATGATCT CTTGAATCATTATCATAAATTC CAGTTTCTGATAGCGG
sh-BTLA-2	GATCCCGGGTCTTCTTCTTAAT CCCATATCTGGACTTCAAGAG AGTCCAGATATGGGATTAAGA AGAAGACCCTTTTTTG	AATTCAAAAAAGGGTCTTCTT CTTAATCCCATATCTGGACTCT CTTGAAGTCCAGATATGGGAT TAAGAAGAAGACCCGG
sh-HVEM-1	GATCCCGTCCACAGTTGGCCT AATCATATGTGTGAATTCAAG AGATTCACACATATGATTAGG CCAACTGTGGATTTTTTG	AATTCAAAAAATCCACAGTTG GCCTAATCATATGTGTGAATCT CTTGAATTCACACATATGATTA GGCCAACTGTGGACGG
sh-HVEM-2	GATCCCGTCCAGGTTATCGTG TGAAGGAGGCCTGCGTTCAA GAGACGCAGGCCTCCTTCAC ACGATAACCTGGATTTTTTG	AATTCAAAAAATCCAGGTTAT CGTGTGAAGGAGGCCTGCGT CTCTTGAACGCAGGCCTCCTT CACACGATAACCTGGACGG

Supplementary Table S3. List of primary antibodies for WB and antibodies for FACS

Antibodies	Manufacturer	Application
BTLA	Abcam, #ab233809	1:1000 for WB
HVEM	Abcam, #ab62462	1:1000 for WB
GAPDH	Proteintech, #60004-1-Ig	1:1000 for WB
p-p44/42 MAPK (p-ERK)	Cell Signaling Technology, #4370S	1:1000 for WB
P44/42 MAPK (ERK)	Cell Signaling Technology, #4695S	1:1000 for WB
APC Mouse IgG1 κ isotype control	BD Biosciences, #554681	1μg/Test for FACS
APC Mouse Anti-Human CD272(BTLA)	BD Biosciences, #564800	1μg/Test for FACS

Reference

1. Gu, Y.; Niu, S.; Wang, Y.; Duan, L.; Pan, Y.; Tong, Z.; Zhang, X.; Yang, Z.; Peng, B.; Wang, X.; et al. DMDRMR-Mediated Regulation of m(6)A-Modified CDK4 by m(6)A Reader IGF2BP3 Drives ccRCC Progression. *Cancer Res* **2021**, *81*, 923-934.
